# Validation of the Tunisian Test for Facial Emotions Recognition: Study in Children From 7 to 12 Years Old

**DOI:** 10.3389/fpsyg.2021.643749

**Published:** 2021-11-22

**Authors:** Amal Taamallah, Soumeyya Halayem, Olfa Rajhi, Malek Ghazzai, Mohamed Moussa, Maissa Touati, Houda Ben Yahia Ayadi, Sami Ouanes, Zeineb S. Abbes, Melek Hajri, Selima Jelili, Radhouane Fakhfakh, Asma Bouden

**Affiliations:** ^1^Hôpital Razi, Manouba, Tunisia; ^2^Faculty of Medicine, Tunis El Manar University, Tunis, Tunisia; ^3^Department of Psychiatry, Hamad Medical Corporation, Doha, Qatar

**Keywords:** social cognition, facial emotional expression, emotion recognition, child, validation study

## Abstract

**Background:** Facial expressions transmit information about emotional state, facilitating communication and regulation in interpersonal relationships. Their acute recognition is essential in social adaptation and lacks among children suffering from autism spectrum disorders. The aim of our study was to validate the “Recognition of Facial Emotions: Tunisian Test for Children” among Tunisian children in order to assess facial emotion recognition in children with autism spectrum disorders (ASD).

**Methods:** We conducted a cross-sectional study among neurotypical children from the general population. The final version of or test consisted of a static subtest of 114 photographs and a dynamic subtest of 36 videos expressing the six basic emotions (happiness, anger, sadness, disgust, fear and surprise), presented by actors of different ages and genders. The test items were coded according to Ekman’s “Facial Action Coding System” method. The validation study focused on the validity of the content, the validity of the construct and the reliability.

**Results:** We included 116 neurotypical children, from 7 to 12 years old. Our population was made up of 54 boys and 62 girls. The reliability’s study showed good internal consistency for each subtest: the Cronbach coefficient was 0.88 for the static subtest and 0.85 for the dynamic subtest. The study of the internal structure through the exploratory factor analysis of the items of emotions and those of intensity showed that the distribution of the items in sub-domains was similar to their theoretical distribution. Age was significantly correlated to the mean of the overall score for both subtests (*p* < 10^–3^). Gender was no significantly correlated to the overall score (*p* = 0.15). High intensity photographs were better recognized. The emotion of happiness was the most recognized in both subtests. A significant difference between the overall score of the static and dynamic subtest, in favor of the dynamic one, was identified (*p* < 10^–3^).

**Conclusion:** This work provides clinicians with a reliable tool to assess recognition of facial emotions in typically developing children.

## Introduction

Social cognition is a central area of social neuroscience, it is a promizing field of exploration of the biological systems that underlie social behavior (Elodie, 2014). SC is a major componant of human adaptation that allows us perceive, process, interpret social information and thus adapt our behavior. It is composed of several domains including theory of mind, empathy, emotional regulation, contextual analysis and facial emotion recognition (FER) (Merceron and Prouteau, 2013; Etchepare et al., 2014). In children, the ability to recognize facial emotions is an important dimension of emotional development. It is built progressively and it depends on cognitive development (Gosselin et al., 1995; Lawrence et al., 2015).

Several studies have developed and validated tests for the evaluation of FER and the rehabilitation of its deficits in adults; however, in children, studies are scarce and present several methodological limits: the lack of standardized measures, and the question of ecological validity (McClure, 2000).

More, heterogeneous results were found about the developmental trajectory of facial emotion recognition and there are only few studies examining this developement in middle and late childhood (Gao and Maurer, 2010; Mancini et al., 2013; Lawrence et al., 2015).

Our work was interested in FER assesment and focused on the study of primary basic emotions, according to Paul Ekman, the pioneer in the field of emotions categorization: happiness, anger, sadness, fear, disgust and surprise as well as neutrality (Golouboff, 2007).

As it has been suggested that facial morphology related to ethnical origin can lead to modulation in emotional facial expression recognition (Gosselin and Larocque, 2000), data with local facial expressions could be more reliable in practice to assess FER (Lee et al., 2013).

The objective of our work was to develop a FER test, entitled « Recognition of Facial Emotions: Tunisian Test for Children » and then to validate it among typically developing Tunisian children, aged between 7 and 12 years old.

This test is part of the Tunisian battery of social cognition assessment which also includes verbal, non-verbal and empathic Tom tasks. The objective is ultimately to develop rehabilitation tools for children with a deficit in social cognition taking into account cultural specificities (Rajhi et al., 2020).

The choice of videos was underpinned by the objective of developing a training tools for children with ASD (Kalantarian et al., 2019).

## Materials and Methods

A cross-sectional study over an 8 month period from November 2018 to June 2019 took place in primary schools and daycare centers in three Tunisian governorates. It was preceded by the development of the digital application.

### Participants

Our population consisted of children aged between 7 and 12 years old, schooled between the 1st and 6th year of ordinary primary school, whose native language is the Tunisian dialect and having a typical development. We did not include children with school failure, and with a present or past psychiatric disorder. Children with school or family troubles were assessed with the MINI K-SADS PL for School-Aged Children-Present and Lifetime Version. Children with intellectual disability, sensory or neurological deficits that interfere with the test performance were excluded from the study.

To assess intelligence, we used the Tunisian version of EDEI-A in its reduced form. We used scale I “vocabulary B” for verbal intelligence and scale VI “categorical analysis” for non-verbal intelligence (Ben Rjeb, 2003).

**FIGURE 1:**
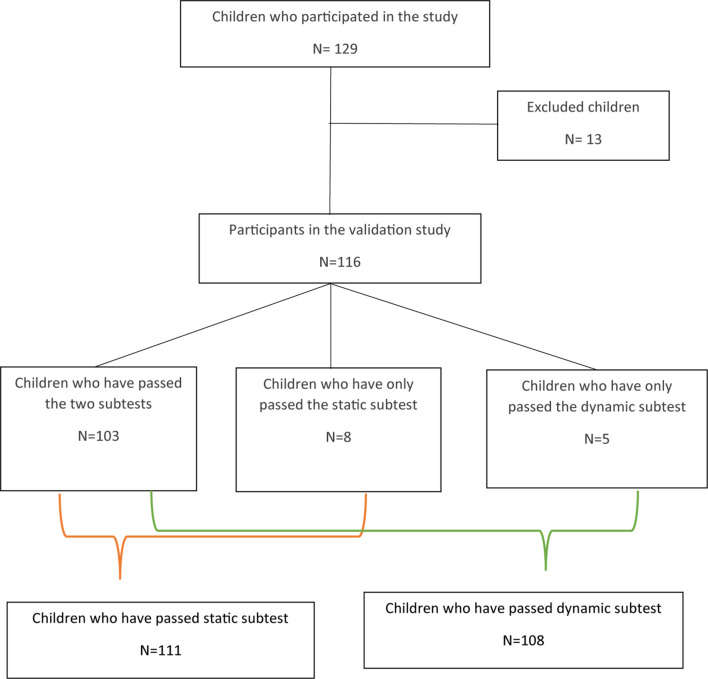
The repartition of participants according to the subtest completed.

After the exclusion of 13 children our sample was made of 116 children. Figure 1 illustrates how many children fulfilled each subtest. Our population was divided into three subgroups according to age: 7–8 years, 9–10 years, and 11–12 years (see Table 1).

**TABLE 1:** Sociodemographic characteristics of the final sample.

Age (years)			9.2 (7–12)
Verbal age on the EDEI-R (years)		Mean (minimum-maximum)	10.4 (6.3–12.6)
Non-verbal age on the EDEI-R (years)			10.7 (5.6–12.3)
Gender (F/M)			53.4/46.6
Age groups (years)	(7–8)	Male/Female/total	23/24/47
	(9–10)		22/17/39
	(11–12)		9/21/30
	Total		54/62/116

### Materials

Our test has been designed as a downloadable application on android. It consists of two subtests: a static subtest and a dynamic one. The static subtest is composed of 114 photographs (18 items per emotion and six items for neutrality), while the dynamic subtest is composed of 36 videos (six items/emotion) as mentioned in the Table 2.

**TABLE 2:** Repartition of the number of photos and videos per emotion.

Emotion	Number of photographs	Number of videos
Happiness	18	6
Anger	18	6
Sadness	18	6
Fear	18	6
Disgust	18	6
Surprise	18	6
Neutrality	6	0
Total	114	36

For its development we were inspired by the Pictures of Facial Affect (POFA) by Ekman and Friesen (1976), Face Test by Baron-Cohen et al. (2001), TREF by Gaudelus et al. (2015), Diagnostic Analysis of Non-verbal Accuracy (DANVA) FACES 2 by Stephen Nowicki (Nowicki and Duke, 2001) and the Test of Recognition of Facial Emotions for Children (TREFE) by Nathalie Golouboff (2007).

Six amateur actors (two adults, two adolescents, and two children), of both sexes, expressed the six basic emotions (happiness, anger, sadness, disgust, fear and surprise). Neutrality has been presented in photographs only.

The photo and video recording sessions took place in a theater club, with a professional photographer.

Each emotion is represented by three intensities (low, medium and intense), for each actor and coded according to Ekman’s method of “Facial Action Coding System.”

The photographs were coded using this latter method, with reference to Gosselin et al. article (Gosselin, 1955) comparing each photograph to that of neutrality. Depending on the system FACS, facial muscle contractions are coded in units of action (AU). The nomenclature includes 46 AU identified by a number. For example, AU1 corresponds to the “inner brow raiser.” For happiness, the units of action involved are AU6 (cheek raiser), AU12 (lip corner puller) and AU 25 (lips part). The intensity of the emotion depends on the number of UA and the intensity of the contraction.

For example, photograph n° 1 is coded d-a-F-3: it is a photograph of disgust (d) of strong intensity [3], represented by an adolescent (a) female (F).

The interface is made up of two parts: the top part displays the photo/video and the bottom part displays the propositions of the emotions in Tunisian dialect, in the form of seven propositions fields to select (6 emotions plus neutrality).

The display of photographs and videos was presented in a random and pre-established order. Each photo is displayed for 15 s and the maximum duration of a video is 5 s (see Supplementary Material 1 for photos and video 1 for an example of video).

For selecting photographs:

Our method was based on a first selection by the experts, followed by a classification by the FACS system then the pilot study. If there was a disagreement between experts, we referred to the majority first and completed the selection of the photos using the FACS system.

The experts initially ruled on 300 photos et 96 videos, eliminating 186 photographs and 60 videos, inexpressive and complex ones. After taking the test, the children appreciated the support and the photographs. Photographs 40, 51, 79, 84, 94, and 96 have been replaced (screenshots from videos) after repeated confusion in responses including complex emotions.

### Procedure

The study protocol was previously approved by the Razi Hospital Ethics comity (see Supplementary Material 2). Then we got the authorization of the headmasters of the primary schools in which the test was taken. Third, the parents of the participants were required to read and sign an informed consent form explaining the aim of the study and the confidentiality of the data. Fourth, participants underwent the pre-test consisting in assessing intelligence and the presence of any psychiatric disorder. Sociodemographic data and the respective pre-test results were input into the “Form” application. Finally, the test was administered individually in an isolated and a quiet room.

The validation study focused on the validity of the content (including experts’ opinion, and pilot study), the validity of the construct (using exploratory factor analysis for the items of each emotion and each intensity) and reliability (using Cronbach’s Alpha).

### Statistical Analysis

Statistical data were input and analyzed using the Statistical Package for Social Sciences (SPSS) program in its 20th version for Windows. Quantitative variables were described using means, standard deviation and limits. Qualitative variables were described using proportions and percentages. We performed a multivariate analysis of variance (MANOVA) to study the difference in mean scores by age group and used Correlation to study test scores according to the gender and age of the children.

For the validation study, we performed an exploratory factor analysis (EFA) with varimax rotation. We used Cronbach’s alpha index to study the internal consistency for each subtest. The significance level chosen was “*p* < 0.05.”

## Results

We will describe the results of validity study and Factors correlated with better performance in REFTTC.

### Validity of Appearance

#### Experts’ Opinion

The photographs and videos were pre-selected by the experts (four child and adolescent psychiatrists (AB, SH, ZA, and MH) and three clinical psychologists (HBY, MT, and HH). The experts initially ruled on 300 photos et 96 videos, eliminating 186 photographs and 60 videos considered as expressive complex and non-basic emotions. Three photographs of increasing intensity for each emotion and each actor were kept.

#### Pilot Study

The pilot study helps to check the understanding of the items by the participants and estimate the time necessary to take the test.

Eighteen typically developing children (nine girls and seven boys) were included.

This step led to replace six photographs (N°40, 51, 79, 84, 94, and 96) due to the repeated confusion in the answers, using screenshots from videos.

### Construct Validity: Exploratory Factor Analysis

#### According to Emotions

Exploratory factor analysis of each emotion of the static subtest, by referring to the eigenvalues greater than 1, made it possible to extract two factors for all the emotions. For the emotion of happiness two factors explained 90.58% of the total variance. For the emotions of disgust, fear, surprise, sadness and anger, two factors explained 86.11, 86.22, 89.99, 83.36, and 84.11% of the total variance, respectively.

However, for the dynamic subtest, one factor explained 94.29% of the total variance, for the emotion of happiness, while for the emotion of surprise, anger, fear, disgust and sadness, one factor explained 84.48, 86.43, 86.43, 87.86, and 80.76%, respectively, of the total variance.

#### According to Intensity

Exploratory factor analysis at the level of the items of each intensity (38 items for each intensity), by referring to the eigenvalues greater than 1, made it possible to extract two factors for the three intensities.

- For the strong intensity, two factors explained 85.04% of the total variance.
- For the average intensity, two factors explained 84.45% of the total variance.
- For the low intensity, two factors explained 81.91% of the total variance.

### Fidelity

#### Internal Coherence

Cronbach’s alpha was 0.88 for photographs and 0.85 for videos.

### Sociodemographic Characteristics of the Sample: Factors Correlated With Better Performance in REFTTC

#### Age

One hundred eleven children took the static subtest: 45 children (40.5%) from the age group of 7–8 years, 37 children (33.4%) from the age group of 9–10 years and 29 children (26.1%) from the 11–12 year age group. The average of correct answers was 76.4/114 (49–97), i.e., 67% (43–85.1%). Correlations between the mean of the overall score and the age was significant and the strength was moderate (*r* = 0.38 and *p* < 10^–3^). The correlation between the mean of the overall score and the age groups (*p* = 0.001) were significant.

One hundred eight children took the dynamic subtest: 44 children (40.7%) from the age group 7–8 years, 36 children (33.3%) from the age class of 9–10 years and 28 children (26%) from the 11–12 age group. The average of correct answers was 28.3/36 (19–35), i.e., 78.7% (52.8–97.2%). The correlation between the overall video score and the age was significant and the strength of the correlation was moderate (*r* = 0.4 and *p* < 10^–3^). The correlation between the score and the age groups showed a significant improvement in the FER with the age (*p* = 0.001).

The comparison between the two means of percentage showed a significant difference (*p* < 10^–^3), with better performance on the dynamic subtest.

#### Gender

On the static subtest, the mean score for boys was 74.7/114 (49–92), and the mean score for girls was 77.7/114 (51–97) with no significant difference (*p* = 0.15) according to gender.

On the dynamic subtest, the mean score for boys was 27.6/36 (19–33) and that of girls was 28.9/36 (20–35) without any significant difference.

The absence of significant difference was also found for the six emotions, according to age.

#### Emotion

##### Static Subtest

**FIGURE 2:**
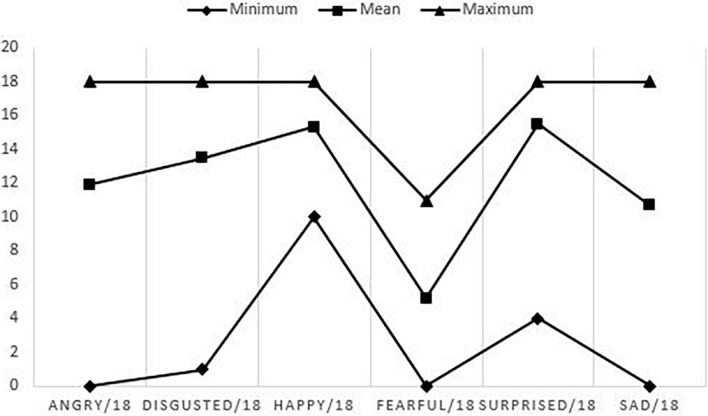
Mean, minimum and maximum of scores according to each emotion on the static subtest.

The mean score according to emotions is shown in Figure 2.

**FIGURE 3:**
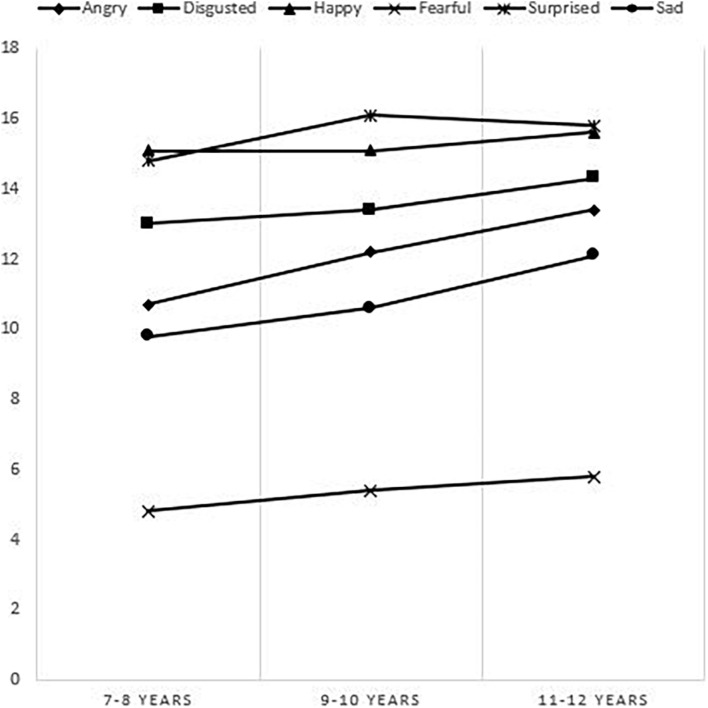
Mean scores per emotion according to age on the static subtest.

The distribution of the mean score by emotion and by age group shows that children between 7–8 years old recognized happiness first, followed by surprise, disgust, anger, sadness and in last fear; while children between 9–10 years old and 11–12 years old recognized surprise first, followed by happiness (Figure 3).

Pearson’s correlation between the following emotions (happiness, surprise, fear and disgust) and age was not significant.

Pearson’s correlation between the following emotions (anger and sadness) and age was significant. The strength of the correlation was moderate for anger (*r* = 0.33 and *p* < 10^–3^) and low for sadness (*r* = 0.26 and *p* = 0.006) (see Table 3).

**TABLE 3:** Pearson’s correlation between the emotions and age on the two subtests of the REF: TTC.

	Emotion	Happiness	Anger	Fear	Sadness	Disgust	Surprise
Static subtest	Age	*p*: 0.53	***P* < 10^–3^ *r*: 0.33**	*p*: 0.6	***p*: 0.006 *r*: 0.26**	*p*: 0.18	*p*: 0.1
Dynamic subtest	Age	*p*: 0.5	***p*: 0.03 *r*: 0.21**	***p*: 0.01 *r*:0.24**	***P* < 10^–3^ *r*: 0.34**	*p*: 0.82	*p*: 0.09

*P values in bold represent significance threshold; r values in bold represent Pearson coefficient.*

##### Dynamic Subtest

**FIGURE 4:**
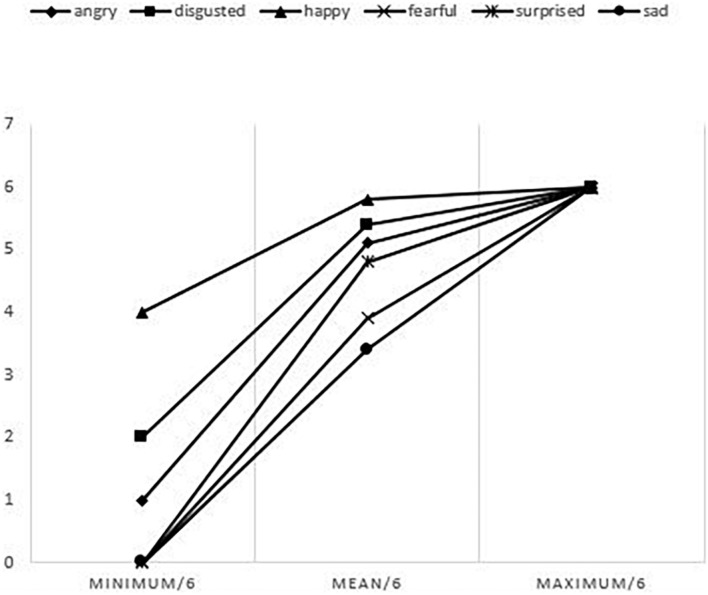
Mean, minimum and maximum of scores according to each emotion on the dynamic subtest.

Figure 4 shows the mean score according to the emotions.

Pearson’s correlations between age and emotions of sadness, anger and fear were significant. The strength of the correlation was moderate for sadness (*r* = 0.34 and *p* < 10^–3^) and low for anger and fear (*r* = 0.21 and *p* = 0.03) (*r* = 0.24 and *p* = 0.01) (see Table 3).

#### Age and Emotion

The distribution of scores for each emotion according to age groups and the result of MANOVA are summarized in the Table 4.

**TABLE 4:** Distribution of the mean score of the emotions of the static and dynamic subtest according to age groups.

	Anger		Disgust		Happiness		Fear		Surprise		Sadness	
	S	D	S	D	S	D	S	D	S	D	S	D
7–8 years	10.7 (0–17)	4.8 (1–6)	13 (1–18)	5.4 (2–6)	15.1 (10–18)	5.8 (4–6)	4.8 (0–9)	3.7 (0–6)	14.8 (4–18)	4.3 (0–6)	9.8 (0–18)	3(0–6)
9–10 years	12.2 (6–17)	5.1 (2–6)	13.4 (2–18)	5.3 (4–6)	15.1 (10–18)	5.8 (4–6)	5.4 (1–11)	3.9 (2–6)	16.1 (13–18)	5.2 (3–6)	10.6 (1–16)	3.6(0–6)
11–12 years	13.4 (5–18)	5.4 (3–6)	14.3 (10–18)	5.4 (4–6)	15.6 (11–18)	5.9 (5–6)	5.8 (2–11)	4.3 (2–6)	15.8 (9–18)	4.8 (1–6)	12.1 (5–16)	4(1–6)
*p*	**<10^–3^**	0.08	0.36	0.78	0.49	0.51	0.2	0.13	0.12	0.01	0.06	0.02

*Bold represent significance threshold.*

The classification of the recognition of facial emotions of the two subtests according to age groups is precised in Table 5.

**TABLE 5:** The classification of the recognition of facial emotions of static and dynamic subtest according to age groups.

Age groups	Static subtest	Dynamic subtest
7–8 years	H > Su > D > A > Sa > F	H > D > A > Su > F > Sa
9–10 years	Su > H > D > A > Sa > F	H > D > Su > A > F > Sa
11–12 years	Su > H > D > A > Sa > F	H > D = A > Su > F > Sa

*H, happiness; Su, surprise; D, disgust; A, anger; Sa, sadness; F, fear. >, better than. =, equal.*

#### Intensity

**FIGURE 5:**
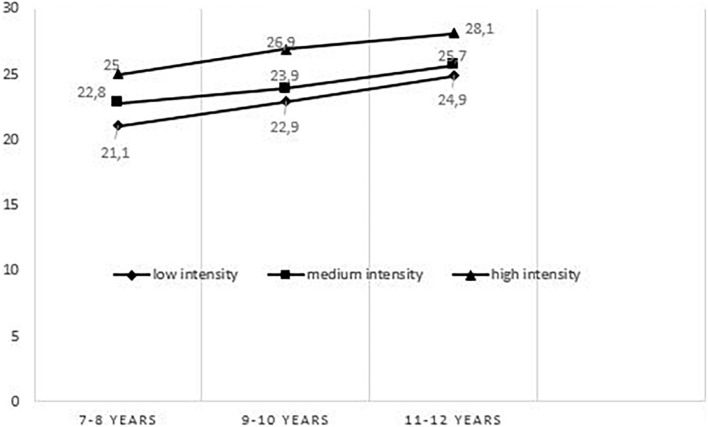
Mean of the static subtest score as a function of intensity and age group.

The mean score by intensity showed that the mean was 22.6/38 (14–31) for low intensity, 23.9/38 (13–31) for medium intensity and 26.4 (17–32) for high intensity (see Figure 5). Pearson’s correlation between age classes and mean intensity scores was significant and the strenght of correlation was moderate for low intensity (*r* = 0.347 and *p* < 10^–3^), medium intensity (*r* = 0.323 and *p* = 0.001) and high intensity (*r* = 0.342 and *p* < 10^–3^).

The comparison between the mean of the scores of the three intensities, for each age group, showed that the strong intensity was better recognized than the medium intensity which was better recognized than the low intensity, with a significant difference (*p* = 0.001 for the age group 7–8 years and 11–12 years, *p* = 0.003 for the age group 9–10 years).

## Discussion

This is a validation study of a new tool of assessment of FER among Tunisian children. This new tool is characterized by static and dynamic assessment of the six6 primary emotions, with three intensities for static emotion assessment of protagonists of the two genders and of three stages of development: child/adolescent and adult. In Table 6 we have summarized the characteristics of our test in comparison of those already proposed in literature.

**TABLE 6:** Comparison between the properties of the tests for the evaluation of facial emotion recognition and our test.

	REF: TTC	Ekman POFA test	DANVA 2	TREFE
**Emotions:** • Happiness • Sadness • Anger • Disgust • Fear • Surprise	+ + + + + +	+ + + + + +	+ + + − + −	+ + + + + −
**Intensities** • One • Many	− + (3)	+ −	− + (3)	+ −
**Children as actor**	+	−	+	+
**Stimuli** • Static • Dynamic	+ +	+ −	+ −	+ −
**Number of participants Age (in years)**	116 (7–12)	−	− (3–99)	279 (7–25)

### Validity of Appearance/Content Validity

The validity of the content is essential in the process of developing a measurement tool. It checks whether the different items of the test fit well into the measurement range (Sireci, 1998). For our work, we studied the comprehensibility and the feasibility of the test. This work was carried out in two stages: the opinion of the experts and the pre-test.

#### Expert Opinion

Experts judged the clarity and relevance of the items, and proposed modifications to improve the comprehensibility and appreciation of the test by children. The experts’ opinion is an important step in cross-cultural validation studies of measurement instruments, thought it is not codified (Streiner et al., 2015).

According to Fermanian, the presence of at least two experts is recommended in order to verify the consistency of their judgments (Fermanian, 2005). Our methodology is comparable to what was described in literature: during the preliminary validation stage of the TREF test, 182 photos were viewed by two experts, who retained 86 photographs according to the FACS criteria (Gaudelus et al., 2015).

#### Pilot Test

Our test was appreciated by the 16 children who found it attractive. Six photographs were removed due to repeated confusions. The use of the pre-test is considered essential by the majority of authors.

It allows to check the understanding of the items by the participants and to estimate the duration of the test (Nowicki and Carton, 1993). The recommended number of participants to be included in a pretest varies between 10 and 40, who must be recruited from the target population for which the instrument is intended (Sousa and Rojjanasrirat, 2011). In the preliminary TREF validation study, the pre-test included a sample of 19 participants and allowed to select 30 photos among the 86 photographs initially selected by the experts (Gaudelus et al., 2015).

### Construct Validity

The assessment of the construct validity of a psychological test consists in the study of its internal and external structures (Vallerand, 1989; Guillemin, 1995). During our validation study, the analysis of the external structure was not possible due to the absence of an already validated test in Tunisia. Hence we assessed only the internal structure by carrying out an exploratory factor analysis (Hair, 2014).

We thus conducted a factorial analysis of the items of each emotion and each intensity. A factorial analysis of the all items in each subtest could not be made given the presence of 114 photographs. The distribution of items in subdomains, determined by factor analysis, was similar to the theoretical distribution of emotions and intensities.

### Fidelity

Fidelity underpins the reproducibility of the results obtained by an instrument (Vaske et al., 2017). The assessment of fidelity focuses on stability and uniformity. Stability concerns the consistency of the results obtained following repeated measurements and it usually refers to the test-retest, which was not carried out in our study since we obtained the agreements for a single assessment.

The second aspect of fidelity is represented by the, measured by the alpha coefficient. The Cronbach’s coefficient was 0.88 for the static subtest and 0.85 for the dynamic one. These two values demonstrate good internal consistency (Nowicki and Carton, 1993).

### Factors Correlated With Better Performance in REF: TTC

#### The Developmental Trajectory of Facial Emotion Recognition in Children

In our work, age was significantly correlated with the total score of correct answers for photographs and videos. Improving the score with age has been shown in the work of Gosselin (Gosselin et al., 1995) and in the validation study of TREFE (Golouboff, 2007). Litterature shows that from the age of three and following the acquisition of language, the child develops the ability to interpret and categorize facial expressions according to the emotion criterion (MacDonald et al., 1996). From the age of 5–6 years, the child becomes able to perform a task of verbal designation of emotions (Gosselin et al., 1995; Vicari et al., 2000). This ability to discriminate is refined with age until and through adolescence (Lenti et al., 1999; Leppanen and Hietanen, 2001). The study by Vicari et al. (2000) examined the development of recognition of emotional facial expressions among 120 children aged 5 to 10 years using a verbal designation of emotions task. Children’s performance increased significantly between the ages of 5–6 and 7–8 years. Also Leppanen and Hietanen (2001) reported a significant improvement in recognition performance between the ages of 7 and 8, 8 and 9, and between the ages of 9 and 10.

Happiness was the most recognized emotion by children for the dynamic stimulus, whatever their age, and by children between 7 and 8 years old, for the static stimulus. For children between 9–10 years old and 11–12 years old, surprise was better recognized than happiness, for the static stimulus. Authors agree on happiness as the first emotion easily recognized by children regardless of their age, the task asked or the stimulus (Boyatzis et al., 1993; MacDonald et al., 1996). In our work, the recognition of the emotion of happiness showed a ceiling effect with average scores for correct answers at 15.1/18; 15.1/18 and 15.6/18, respectively, for the three age groups (Figure 3). Our results are comparable to those of Lawrence et al. (2015) who assessed children from 6 to 16 years.

Sadness was influenced by age in our simple as it was found in literature in comparable age groups (Gosselin et al., 1995; Vicari et al., 2000; Golouboff, 2007; Lawrence et al., 2015).

Anger recognition was correlated to age in our sample. This was also reported by Vicari et al. (2000) and TREFE (Vicari et al., 2000; Golouboff, 2007) for a relatively comparable age groups sample. However, Lawrence et al. (2015) found that pubertal status influenced predominantly this competence.

Though enhancing slightly, fear recognition was not significantly correlated to age in our sample for the static subtest. Our data seem to be in accordance with literature who found slow increase of this performance for this age without controlling for its statistic significance (Vicari et al., 2000).

Disgust and surprise recognition was also not significantly modified by chronological age in school age children (Vicari et al., 2000).

Vicari et al. (2000) concluded that « Generally, children’s performance improves with age, and this is especially clear for sadness, anger and fear. It may well be that the relatively flat developmental profiles for happiness, disgust and surprise reflect ceiling effects for these particular facial expressions.

Some studies have shown that school-aged children recognize the expression of anger better than those of sadness, surprise and fear (Leppanen and Hietanen, 2001). Others concluded that the recognition of facial expressions of happiness, sadness and anger was better than that of fear, surprise and disgust (Boyatzis et al., 1993; Vicari et al., 2000). Still others reported that faces expressing disgust were better identified than those expressing anger, sadness or fear (Lenti et al., 1999).

First, surprisingly, according to all these studies, children seem to have difficulty recognizing facial expressions of fear. Since facial expressions of fear and surprise share many of the same characteristics (large eyes, open mouth, raised eyebrows), the children may confuse the two. This type of confusion has often been observed in children, as in adults (Ekman and Friesen, 1971; Gosselin et al., 1995; Gosselin and Larocque, 2000; Rapcsak et al., 2000).

Second, the discrepancies in accuracy of FER according to emotion can be partly explained by the difference between the nature of the stimuli (photographs of Ekman, photographs of children and schematic faces) and the nature of the task requested (pointing, matching or verbal designation). Vicari et al. (2000) also demonstrated that the trajectory of FER development differed according to the cognitive component involved in the task which is determined by the type of the task –forced choice or not protocol- and the number of propositions presented to the child (Vicari et al., 2000; Lancelot et al., 2012). In their study, happiness, was followed by sadness, anger, fear, surprise and disgust, during the pointing task. For the task of verbal designation, happiness and sadness were followed by surprise, disgust, anger and fear.

### The Effect of Gender

We did not find significant differences according to gender. Mc Clure, in a meta-analysis published in 2000 covering 104 studies, shows a slight female advantage. This benefit is important in babies, then decreases in preschoolers to become stable in childhood and adolescence (McClure, 2000; Thayer and Johnsen, 2000). Other studies have found no difference between FER performance in girls and boys (Calvo and Lundqvist, 2008; Joyal et al., 2014).

### The Effect of Intensity

We found that children had better scores for high intensity photographs followed by those of medium intensity then those of low intensity. This result is overall in agreement with the data in the literature (Herba et al., 2006; Orgeta and Phillips, 2007; Gao and Maurer, 2009).

### The Effect of the Static or Dynamic Nature of the Test

Significant better scores were found for dynamic subtest in comparison to static subtest (*p* = 0.00). The study by Joyal et al. (2014) compared dynamic virtual stimuli with animations developed from POFA stimuli. Two types of stimuli were similarly recognized by adults. The authors highlighted the advantages of avatars which, in addition to being dynamic, can be modified and personalized to meet the specific needs of the studies. Virtual reality allows indeed an increase in ecological validity (Joyal et al., 2014; Cigna, 2015). The absence of a significant difference between the recognition of avatars and photographs can be explained by the age of the participants (adults) or by the nature of the dynamic stimulus. These results lead us to think of the importance of developing other tests for the evaluation and/or re-education of the field of FER, while approaching reality *via* the integration of voice, body language and the environment generating emotion (Tardif et al., 2007; Lainé et al., 2011).

During our work we detected difficulties that we took into consideration to improve our test. The sample of our study was small and requires an enlargement of the population in order to be able to compare the subgroups and draw conclusions.

## Conclusion

Our test was deemed valid with the development of normative references for the age group from 7 to 12 years old. We found satisfactory results of the validity of the REF: TTC test. However, a suggestion to eliminate two photographs of each intensity and each emotion is proposed to alleviate the test. This validation work is a first step which must be completed by an enlargement of the sample, by the test retest procedure and by a validation study in the clinical population. This will allow us to refine our results in order to propose the test as a well-coded clinical evaluation tool and in a second step as a basis for training FER among children with ASD.

## Data Availability Statement

The raw data supporting the conclusion of this article will be made available by the authors, without undue reservation.

## Ethics Statement

Written informed consent was obtained from the individual(s), and minor(s)’ legal guardian/next of kin, for the publication of any potentially identifiable images or data included in this article.

## Author Contributions

AT: elaboration of the test and its administration, statistical analysis, and redaction of the article. SH: elaboration of the test, statistical analysis, and correction of the article. OR, MG, and MM: administration of the test. SO: English revision. ZA, MH, and SJ: elaboration of the test. HA and MT: choice of the pre-test. RF: elaboration of the research protocol, statistical analysis, and correction of the article. AB: elaboration of the test and the research protocol and correction of the article. All authors contributed to the article and approved the submitted version.

## Conflict of Interest

The authors declare that the research was conducted in the absence of any commercial or financial relationships that could be construed as a potential conflict of interest.

## Publisher’s Note

All claims expressed in this article are solely those of the authors and do not necessarily represent those of their affiliated organizations, or those of the publisher, the editors and the reviewers. Any product that may be evaluated in this article, or claim that may be made by its manufacturer, is not guaranteed or endorsed by the publisher.
