# Supplementary figures and images for: Validation of the Tunisian Test for Facial Emotions Recognition: Study in Children From 7 to 12 Years Old
Source: Front Psychol. 2021 Nov 22;12:643749. doi: 10.3389/fpsyg.2021.643749 (PMC8645551; doi:10.3389/fpsyg.2021.643749)

Exemples of the interface and the different age of the actors


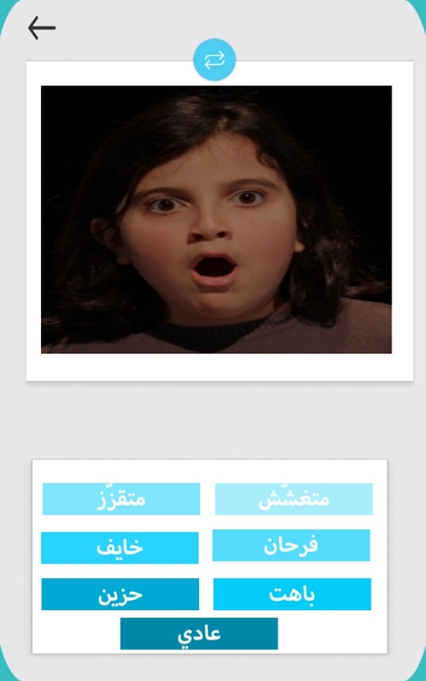


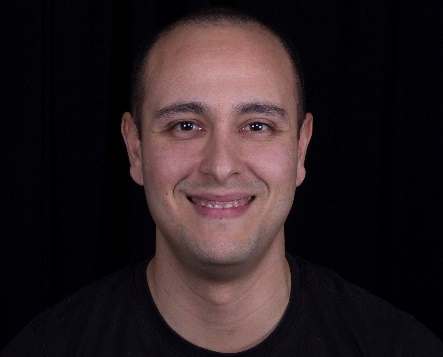

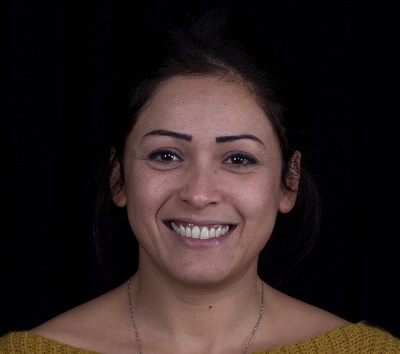

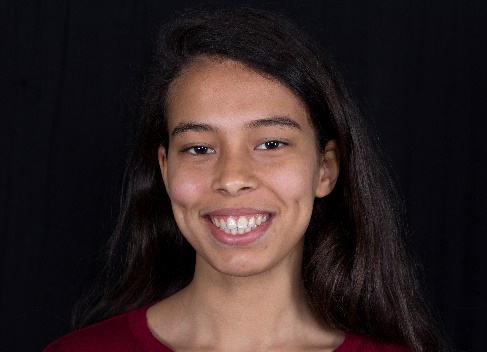

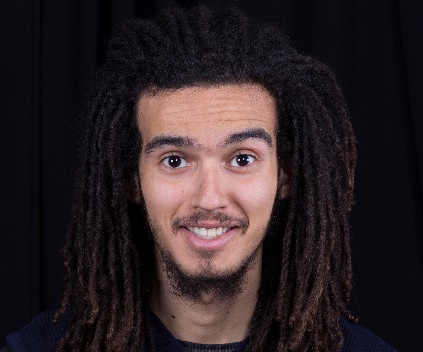

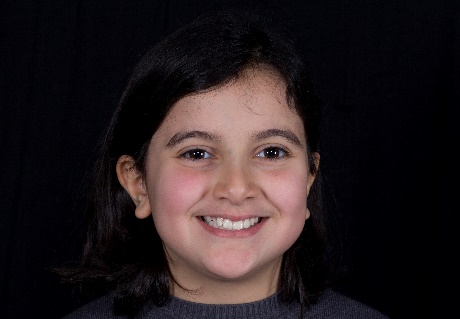

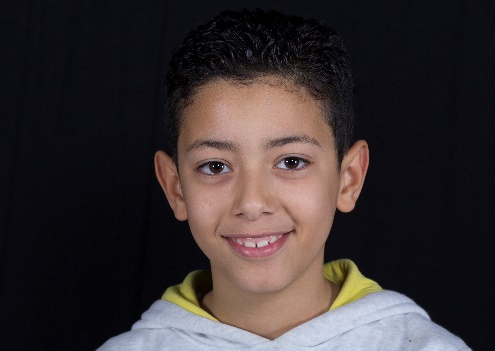

Supplement: Supplementary file 1 [file Table_1.DOCX]

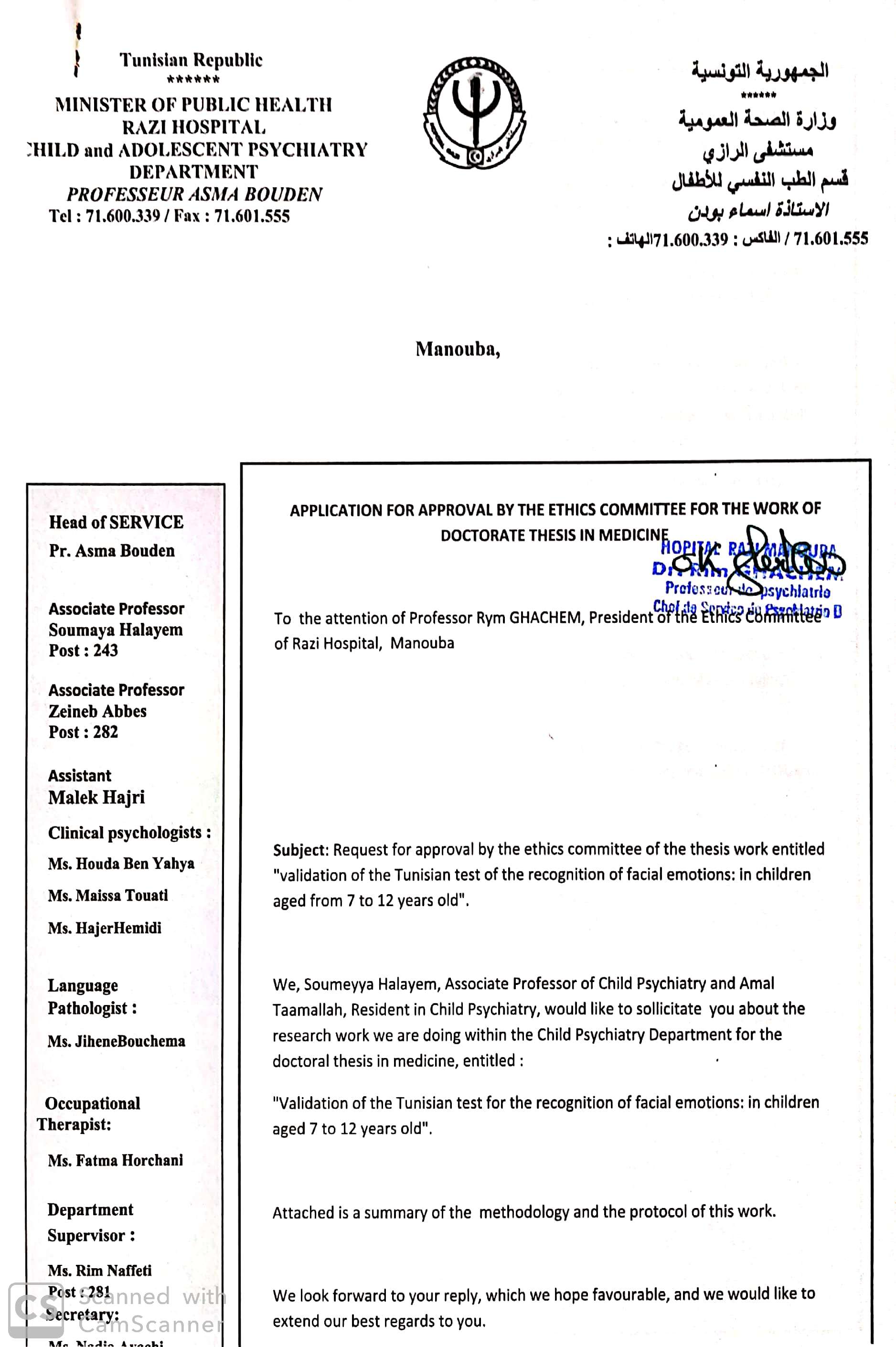


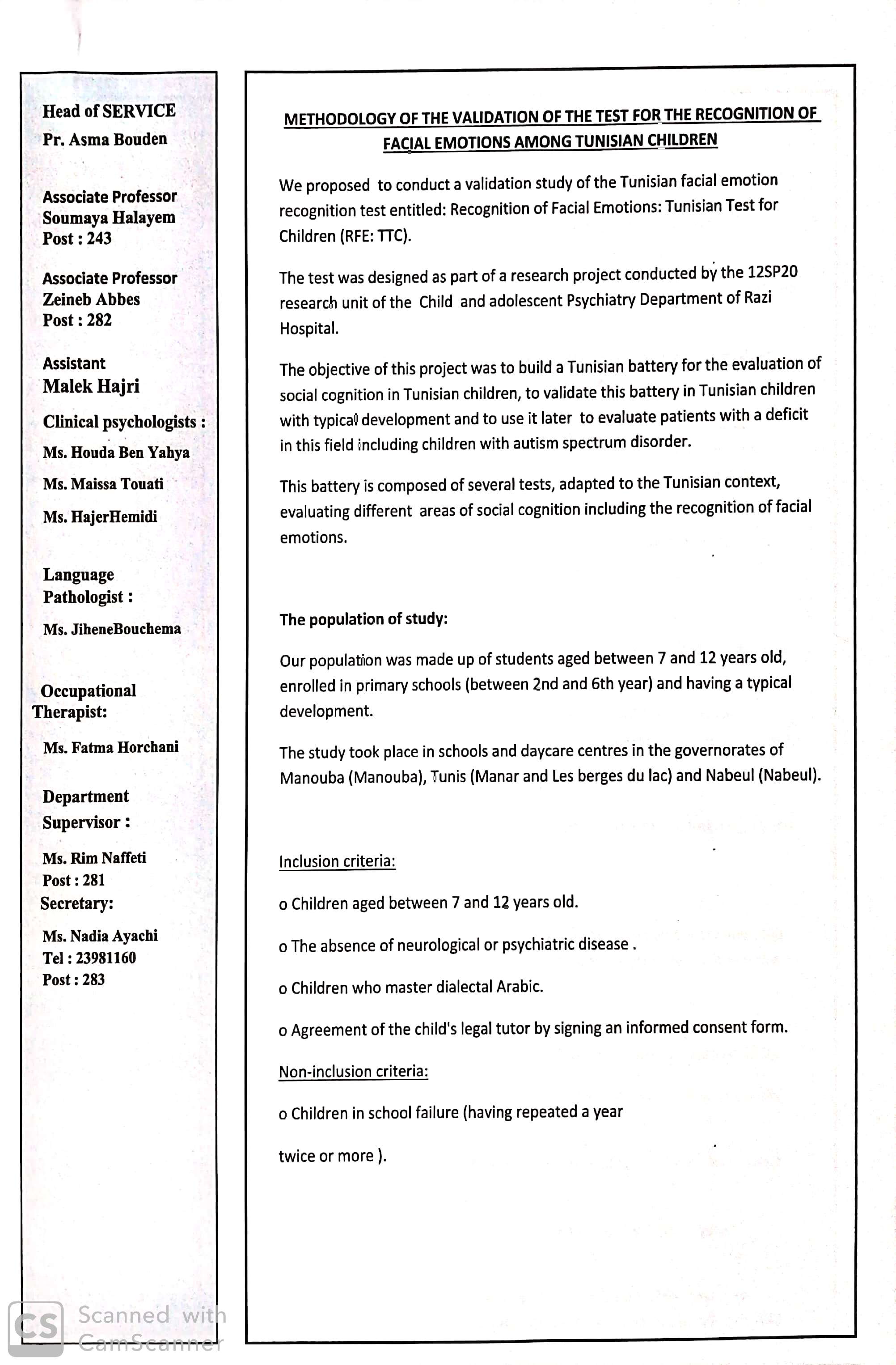


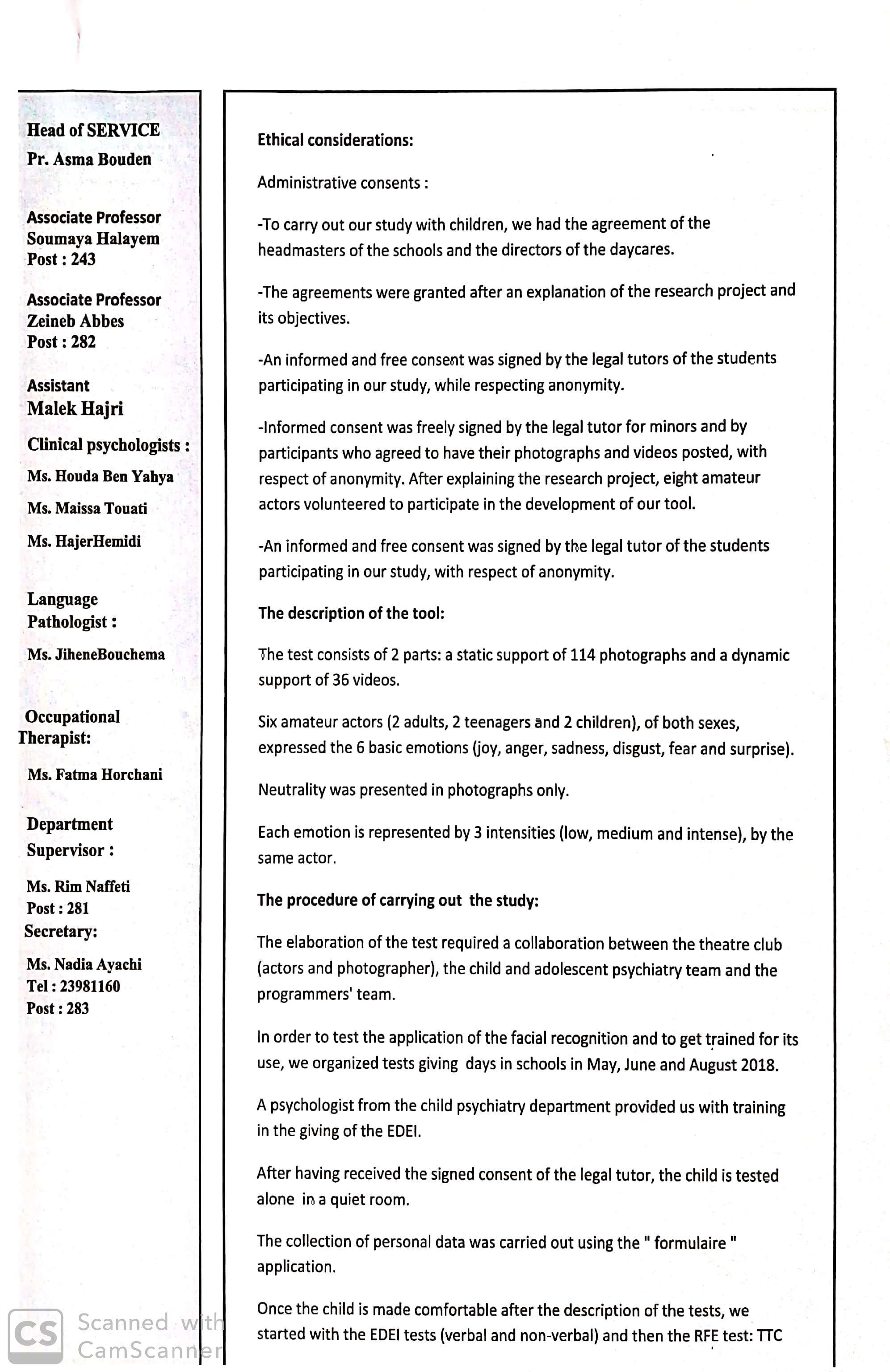


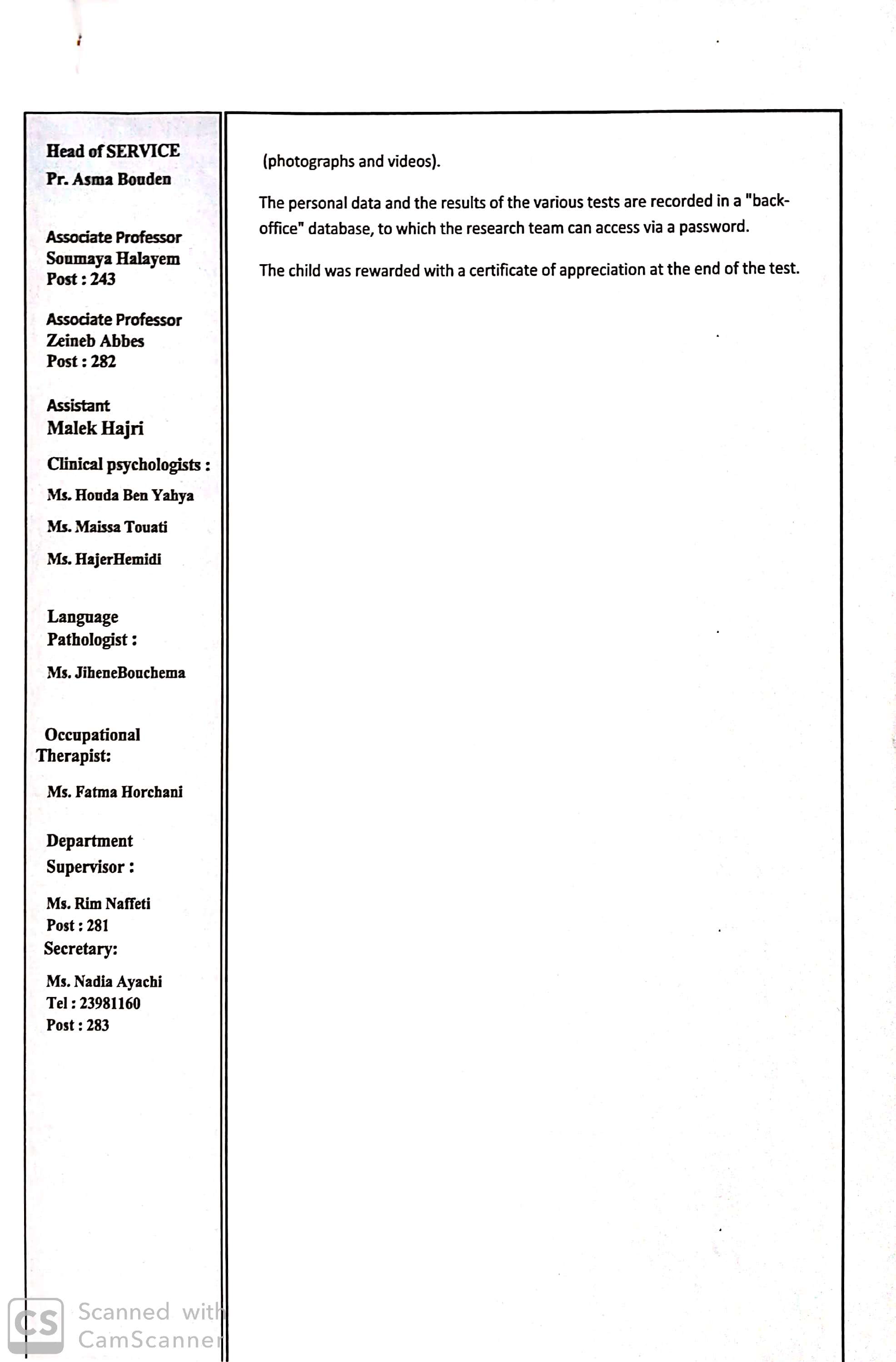

Supplement: Supplementary file 2 [file Table_2.DOCX]
